# Knowledge of dental photography among dentists: A cross-sectional study

**DOI:** 10.6026/973206300214088

**Published:** 2025-11-15

**Authors:** Balaji V.R, Arunmozhi U, Manikandan D, Jane Carolyn Maansingh

**Affiliations:** 1 Department of Periodontics & Implant Dentistry, CSI College of Dental Sciences and Research, Madurai, Tamil Nadu, India; 2 Department of Periodontology and Oral Implantology, Sri Venkateswara Dental College and Hospital, Chennai, Tamil Nadu, India

**Keywords:** Dental photography, dental students, dentists, clinical photography

## Abstract

Clinical dental photography has become an integral part of dentistry, aiding documentation, patient education, treatment planning and aftercare. With advances in digital technology, both students and practitioners widely use dental photography, significantly influencing clinical practice. Therefore, it is of interest to assess the extent of use, knowledge and ethical perceptions of dental photography among dental practitioners and students through a structured questionnaire survey. An observational cross-sectional online survey was conducted and statistical analysis revealed no significant differences in knowledge between experienced clinicians and students. While dental photography is widely adopted for documentation and patient motivation, there remains a need to strengthen technical skills and ethical awareness to optimize its role in practice.

## Background:

Photography has become an indispensable tool in every facet of life. It is now a powerful tool for communication and expression. As patient demands for aesthetics develop, the use of this technology in dentistry is distinguished by its simplicity, speed and exceptional effectiveness in clinical research, patient education and the documenting of procedural work. Consequently, it provides numerous advantages for both patients and dentists [1, 2]. In 1840, the world's first dental school opened the first photographic gallery, marking the beginning of the history of dentistry with photography. The historical connection between these two disciplines started with this establishment, which was run by a dentist who also became a photographer [6]. As time has gone on, photography has become an essential aspect of patient records and treatment planning, strengthening the long-standing relationship between dentistry and photography. Due to its many advantages, photography is a valuable clinical, administrative and marketing tool. These advantages include: i) Helping with diagnosis and treatment plan development; ii) Improving patient education in dentistry; iii) Motivating and inspiring patients; iv) Facilitating efficient communication with insurance companies, laboratories and colleagues; v) Facilitating referrals to specialists; vi) Serving medico-legal purposes; and vii) Promoting advertising and marketing initiatives [3, 5]. To make sure patients agree to the usage of these photos, it is best to get their written agreement. By improving dental treatment and services, these methods help to raise the standard of care provided. Sharland *et al.* claim that dentists typically overlook clinical photography because of a lack of time, inexperience with photography, unavailable internet and costly equipment [3]. Digital photography's disadvantages are typically the initial investment of funds and the constantly changing technological landscape, which creates an initially challenging and sometimes daunting learning curve [7]. However, it should be noted that clinical photography is no more frightening than many other clinical procedures. Therefore, it is of interest to evaluate dental students' and dentists' clinical practice understanding and application of dental photography.

## Materials and Methods:

To learn more about the use and awareness of dental photography among Tamil Nadu dentists and dental students, an anonymous web-based survey was used. After providing the necessary consent, 130 individuals completed the questionnaire. Thirty questions about the use, scope and future prospects of dental photography were included in the questionnaire.

## Results:

A total of 130 individuals responded out of 136. Thus, 94.9% was the overall response rate. Of the 130 responders, 24 were men and 106 were women. Of the respondents, 54 were dental students, 31 were interns, 27 were general practitioners and 18 were specialists. The most popular tool for taking dental photos was a phone camera (77.6%), which was followed by a DSLR camera (22.4%) (Figure 1 - see PDF). The most necessary prerequisite for shooting photos, according to survey respondents, was documentation (67.3%), followed by study/research reasons (51.4%) and treatment result monitoring (63.6%) (Figure 2 - see PDF). Almost 89.7% of participants obtained consent before taking pictures of patients and all groups were aware that consent was required. However, just 8% of them obtained written consent, 12.1% obtained both and the majority required verbal consent (75.7%). Just 10.3% of respondents had previously taken dental photography courses, with 61.7% of respondents having never taken any (Figure 3 - see PDF). Participants were tested on their fundamental understanding of DSLRs. Just a small percentage of participants showed accurate understanding in their responses, while the majority lacked basic information of DSLR cameras (Figure 4a - see PDF, 4b - see PDF, 4c - see PDF). Participants' perceptions on clinical photography's future prospects were assessed. The majority (97.7%) thought that using clinical photography improved clinical efficiency and 92.3% thought that present dental photography procedures might be improved. 90% also thought that dental photography-related seminars, workshops, or continuing education (CDE) sessions ought to be a regular part of their curriculum. Furthermore, 39.2% of respondents said they would be willing to take proactive measures to advance their skills, while 56.9% said they are already doing so (Figure 5 - see PDF).

## Discussion:

One notable development that has resulted from the integration of technology in the realm of modern dentistry is dental photography. These pictures are useful resources for diagnosis, treatment planning, patient communication and recording treatment results [9]. The results of this poll showed certain tendencies among respondents. Although a sizable fraction of dental practitioners showed a rudimentary comprehension of dental photography, there was considerable variation in the breadth of their knowledge and practical use. According to Abouzeid *et al.* dentistry students frequently utilise mobile cameras to take pictures of their patients. This is consistent with the findings of the current study, which found that the majority of participants used mobile phones for photography [1]. Despite their relatively recent emergence as intraoral photography equipment, DSLR cameras and DSLRs with macro lenses were the next most popular devices behind mobile phones. This rapid uptake of cutting-edge technology, however, might be a result of people realising how easy it is to store digital images on computers and not having to develop, print, or utilise slides. This is consistent with Sharland *et al.*'s findings, which also pointed out how quickly DSLR cameras equipped with macro lenses are being used in clinical photography [4]. Ferreira *et al.* suggested a basic photographic protocol using mobile devices for both intraoral and extraoral images, emphasizing accessibility for dental students and clinicians. Their review concluded that although DSLR cameras remain the gold standard, modern smartphones can produce diagnostically acceptable images when correct lighting, angulation and positioning are followed. This aligns with the present study's findings that a majority of practitioners rely on mobile devices, underscoring the need for formal training on smartphone-based dental photography to improve image quality and standardization [11]. Ensuring patient anonymity and permission is a major concern in clinical photography. Similar to this, our survey found that a large number of participants strongly thought that the dentistry curriculum needed specialised courses, programs, or even just such tools to help practitioners become more skilled and knowledgeable. In line with Hannah *et al.*'s findings, the majority of participants had never taken any dental photography courses before [10]. The report did, however, also point out several concerning areas. Both students and dentists who participated in the study had a significant lack of thorough knowledge of sophisticated camera settings like ISO, shutter speed and aperture, which are essential for taking crisp, correct photos. Albugami *et al.*'s findings are comparable to this one [8]. In a 2025 cross-sectional study, Alqabbani *et al.* demonstrated that dental photography shared on social media platforms significantly influences patients' choice of dentists, particularly among younger and female populations. The study found that before-and-after clinical images enhanced patient trust and treatment acceptance. These findings expand the scope of dental photography beyond clinical documentation, highlighting its role in patient communication, marketing and reputation building in the digital era [12]. Similarly, Cheng Han Lee *et al.* conducted a cross-sectional assessment among dental students in Malaysia and reported a comparable lack of comprehensive knowledge regarding clinical photography. Their findings reinforced the importance of integrating structured training modules within the dental curriculum to ensure competence in both technical and ethical aspects of dental imaging [13].

## Conclusion: 

This cross-sectional survey emphasizes the need for better dental photography training in dental schools and continuing education programs. Strengthening theoretical and practical skills will help practitioners use photography effectively for diagnosis and treatment planning. Proper training also enhances communication with patients and supports professional growth. Bridging the knowledge gap is essential to maintain high standards of patient care. Continuous learning will further encourage innovation in this rapidly evolving aspect of modern dentistry.
